# Engineering of extracellular matrix from human iPSC-mesenchymal progenitors to enhance osteogenic capacity of human bone marrow stromal cells independent of their age

**DOI:** 10.3389/fbioe.2023.1214019

**Published:** 2023-08-02

**Authors:** Dominik Hanetseder, Tina Levstek, Andreas Herbert Teuschl-Woller, Julia Katharina Frank, Barbara Schaedl, Heinz Redl, Darja Marolt Presen

**Affiliations:** ^1^ Ludwig Boltzmann Institute for Traumatology, The Research Centre in Cooperation with AUVA, Vienna, Austria; ^2^ Austrian Cluster for Tissue Regeneration, Vienna, Austria; ^3^ Department Life Science Engineering, University of Applied Sciences Technikum Wien, Vienna, Austria; ^4^ University Clinic of Dentistry, Medical University of Vienna, Vienna, Austria

**Keywords:** extracellular matrix, iPSCs, bone marrow stromal cells, aging, osteogenic differentiation

## Abstract

Regeneration of bone defects is often limited due to compromised bone tissue physiology. Previous studies suggest that engineered extracellular matrices enhance the regenerative capacity of mesenchymal stromal cells. In this study, we used human-induced pluripotent stem cells, a scalable source of young mesenchymal progenitors (hiPSC-MPs), to generate extracellular matrix (iECM) and test its effects on the osteogenic capacity of human bone-marrow mesenchymal stromal cells (BMSCs). iECM was deposited as a layer on cell culture dishes and into three-dimensional (3D) silk-based spongy scaffolds. After decellularization, iECM maintained inherent structural proteins including collagens, fibronectin and laminin, and contained minimal residual DNA. Young adult and aged BMSCs cultured on the iECM layer in osteogenic medium exhibited a significant increase in proliferation, osteogenic marker expression, and mineralization as compared to tissue culture plastic. With BMSCs from aged donors, matrix mineralization was only detected when cultured on iECM, but not on tissue culture plastic. When cultured in 3D iECM/silk scaffolds, BMSCs exhibited significantly increased osteogenic gene expression levels and bone matrix deposition. iECM layer showed a similar enhancement of aged BMSC proliferation, osteogenic gene expression, and mineralization compared with extracellular matrix layers derived from young adult or aged BMSCs. However, iECM increased osteogenic differentiation and decreased adipocyte formation compared with single protein substrates including collagen and fibronectin. Together, our data suggest that the microenvironment comprised of iECM can enhance the osteogenic activity of BMSCs, providing a bioactive and scalable biomaterial strategy for enhancing bone regeneration in patients with delayed or failed bone healing.

## 1 Introduction

Bone has a natural ability for regeneration and most bone defects heal successfully after the restoration of alignment and stable fixation. However, when natural bone healing is impaired, surgical treatment with grafting is needed (Fröhlich et al., 2008). Extensive tissue damage with compromised vascular supply, advanced patient age and associated diseases present major risk factors for delayed or impaired bone healing (Clark et al., 2017). During aging, bone grows more fragile and is less able to perform its mechanical function and calcium reservoirs are often depleted (Boskey and Coleman, 2010). Furthermore, a shift in bone marrow from red to fatty yellow marrow (Rozman et al., 1989) and a decline in the number and regenerative potential of bone progenitor cells negatively impact bone regenerative capacity (Gibon et al., 2016; Kassem and Marie, 2011). Due to the limitations of current treatments, including the inert nature of commonly used implant materials, mesenchymal stromal cells (MSCs) from bone marrow (BMSCs) and other sources have been extensively studied for cell therapies and tissue engineering (Fröhlich et al., 2009; Martin et al., 2019). However, MSCs themselves are subject to aging-related changes, including decreases in stem cell quantity, general fitness, proliferation and osteogenic differentiation capabilities, as well as alterations in gene expression and signaling profiles (Zupan et al., 2021).

In native tissues, mesenchymal cells are surrounded by an extensive extracellular matrix (ECM) composed of structural proteins such as collagens, fibronectin, elastin, glycosaminoglycans, and proteoglycans (Gordon, 1988; Hocking et al., 1998; Nilsson et al., 1998; McKee et al., 2019). The ECM plays an important role in regulation of wound healing, angiogenesis, adipogenesis, fibrosis, autophagy, tumor growth, and metastasis (Monboisse et al., 2014; Ricard-Blum and Vallet, 2016a; Ricard-Blum and Vallet, 2016b). On the cellular level, it modulates cell signaling, gene expression, and enzyme activity (Genovese and Karsdal, 2016; Ricard-Blum and Vallet, 2019). It was previously shown that *in vitro* engineered ECM can maintain proliferation, stemness, and osteogenic differentiation of human BMSCs better than standard tissue culture plastic (Lai et al., 2010; Lin et al., 2012; Zhang et al., 2016). It is known that osteoprogenitor cells adhere to ECM proteins via cell surface integrins, and the integrin binding affects attachment and differentiation of those cells (Nieto-Nicolau et al., 2020; Schwab et al., 2013). ECM produced by young adult (20–30 years) human BMSCs has previously been engineered into three-dimensional (3D) collagen/hydroxyapatite and polycaprolactone scaffolds and had favorable effects on young human BMSC proliferation, stemness, and osteogenic differentiation potential (Antebi et al., 2015; Silva et al., 2020). Similarly, hybrid material comprised of decellularized mineralized human BMSC-ECM in polyurethane scaffolds, re-seeded with human BMSCs, enhanced their osteogenic differentiation *in vitro* and *in vivo* (Sadr et al., 2012). ECMs deposited by human umbilical cord MSCs and mouse BMSCs into 3D scaffolds also promoted M2 macrophage accumulation, hematopoietic stem cell (HSC) adhesion, and MSC-HSC interaction, thereby enhancing bone regeneration (Deng et al., 2020; Lai et al., 2013).

The chronological age of ECM-producing cells was shown to affect the functional properties of ECM to some extent (Carvalho et al., 2021). In a rat model, Sun et al. showed that ECM engineered from young BMSCs could enhance replication and osteogenic differentiation of aged BMSCs, whereas ECM derived from aged BMSCs could not (Sun et al., 2011). Human fetal femur BMSC-derived ECM improved expansion and preserved multipotency of adult BMSCs at higher levels than the ECMs derived from adult BMSCs (Ng et al., 2014). In this study, enhancement of cell proliferation and differentiation was presumably due to higher ECM yields and growth factors deposition by fetal BMSCs (Ng et al., 2014).

However, primary human MSCs face several limitations that make their use in engineering and production of ECM for clinical applications challenging. MSCs are extremely rare in the bone marrow (approx. 0.01%–0.001% of all nucleated cells) (Friedenstein et al., 1982; Wexler et al., 2003). Despite their higher abundance in some other tissues, such as adipose tissue, the use of MSCs is limited by their finite *in vitro* expansion potential and significant variation in their properties dependent on sources of origin. Adult MSC´s regenerative potential declines with age and extended culture, and the use of fetal human MSCs is limited due to ethical issues (Zupan et al., 2021; Frobel et al., 2014; Zhang et al., 2009). Furthermore, MSCs can reduce the secretion of factors important for tissue regeneration, such as hepatocyte growth factor (HGF) or interleukin-1β, during expansion *in vitro*, thus precluding their therapeutic use (Lian et al., 2010; Mabotuwana et al., 2022).

In contrast, human induced pluripotent stem cells (hiPSCs) represent a scalable source for deriving unlimited amounts of standardized human MSC-like mesenchymal progenitors (hiPSC-MPs) (Frobel et al., 2014; Lian et al., 2010; De Peppo et al., 2013). Furthermore, cellular reprogramming to hiPSCs allows for epigenetic rejuvenation of the adult cells, thus resulting in mesenchymal progenitors with youthful characteristics (Frobel et al., 2014; Lapasset et al., 2011). It was previously shown that hiPSC-MPs closely resemble embryonic stem cell-derived mesechymal progenitors in their global gene expression profile and have a higher proliferation potential than the young adult BMSCs (De Peppo et al., 2013; Sheyn et al., 2016). Thus, hiPSC-MPs represent a promising human cell source for ECM engineering and eventual clinical translation.

To our knowledge, the potential of ECM engineered from human iPSC-derived mesenchymal progenitors (iECM) to enhance osteogenic capacity of primary human BMSCs from donors of various ages has previously not been assessed. In this study, we evaluated iECM as a layer deposited on standard two-dimensional (2D) tissue culture dishes and as a coating in 3D spongy silk fibroin scaffolds. Silk fibroin was chosen as the supporting scaffold due to its favorable mechanical properties, biocompatibility, and prior studies demonstrating its potential for bone tissue engineering (Marolt et al., 2006; Deininger et al., 2021; Sun et al., 2021). We hypothesized that iECM would enhance the osteogenesis of young adult BMSCs and partially restore the impaired osteogenesis of aged BMSCs, which is an unsolved challenge in standard osteogenic differentiation models on tissue culture plastic. We also compared the effects of iECM to the effects of ECMs from young adult- and aged BMSCs, and tested whether the iECM has a stronger effect on osteogenic differentiation as compared to single protein substrates.

## 2 Materials and methods

### 2.1 Materials

Gelatin, tissue culture water, Dulbecco’s modified Eagle’s medium high glucose (DMEM-HG), fetal bovine serum (FBS), penicillin-streptomycin (P/S), L-glutamine, ascorbic acid-2-phosphate (A2P), dexamethasone, ß-glycerophosphate, Triton X-100, ammonia solution (NH_4_OH), DNase I, p-nitrophenol, 2-amino-2-methyl-1-propanol, magnesium chloride (MgCl_2_), para-nitrophenyl phosphate (pNPP), sodium hydroxide (NaOH), DNA standard, sodium chloride (NaCl), sodium citrate dihydrate, ethylenediaminetetraacetic acid (EDTA), L-cysteine, L-papain, Hoechst 33,342, Alizarin red S, sodium carbonate (Na_2_CO_3_), lithium-bromide (LiBr), hexafluoroisopropanol (HFIP), methanol, trichloroacetic acid, and formaldehyde were purchased from Sigma-Aldrich (St. Louis, United States). HyClone FBS, dialysis tubing, KnockOut Dulbecco’s modified Eagle’s medium (KO-DMEM), GlutaMAX, nonessential amino acids, and ß-mercaptoethanol were from Fisher Scientific (Pittsburgh, United States). Basic fibroblast growth factor (b-FGF) and DNase I were from Invitrogen (Fisher Scientific, Pittsburgh, United States). Triton X-100 and sodium phosphate monobasic monohydrate (NaH_2_PO_4_ * 1 H_2_O) were from Fluka (Honeywell Research Chemicals, Morris Plains, United States), and phosphate-buffered saline (PBS) without Ca^2+^ and Mg^2+^ was from Lonza (Basel, Switzerland). All other chemicals were purchased from Sigma-Aldrich (St. Louis, United States), unless stated otherwise.

### 2.2 Fabrication of porous 3D silk scaffolds

Porous silk fibroin scaffolds were prepared from silkworm (*Bombyx mori*) cocoons as previously described (Teuschl et al., 2017). Briefly, the silk sericin was extracted by boiling in 0.02 M Na_2_CO_3_ solution for 60 min, and the raw silk fibroin was dissolved in 9.3 M LiBr solution at 60°C for 4 h. Afterwards, the solution was dialyzed for 48 h against distilled water to remove unwanted salt ions and the resulting silk fibroin solution was centrifuged for 15 min at 4700 × g at 4°C, lyophilized, and stored at room temperature (RT). For porous scaffolds preparation, NaCl was first sieved with a metal mesh to obtain particles sized between 300 and 500 µm. 15% silk fibroin solution was prepared in HFIP, mixed 1:1 with salt particles and casted into disc-shaped containers. The containers were incubated at 37°C for 24 h, then the silk was treated with 90% methanol for 30 min to induce beta sheet formation. Finally, the silk/NaCl blocks were immersed in distilled water for 2 days to remove NaCl. The resulting porous silk scaffolds were cut into cylinders of 8 mm diameter and 2 mm thickness and stored in 70% EtOH at RT prior cell culture.

### 2.3 Cell culture

The hiPSC-MPs generated and characterized in detail in our previous study (De Peppo et al., 2013) were expanded in KO-DMEM supplemented with 20% HyClone FBS, 100 U/mL P/S, 2 mM GlutaMAX, 0.1 mM nonessential amino acids, 0.1 mM β-mercaptoethanol, and 1 ng/mL b-FGF. Cells were seeded on standard cell culture flasks pre-coated with 0.1% (wt/v) gelatin in tissue culture water and cultured in a humidified incubator at 37°C and 5% CO_2_ until confluency. Cells of passages 9 to 11 were used for iECM production.

BMSCs were expanded in medium consisting of DMEM-HG supplemented with 10% FBS, 100 U/mL P/S, 2 mM L-glutamine and 1 ng/mL b-FGF and characterized according to the consensus position statement of the International Society for Cellular Therapy (Dominici et al., 2006) (data not shown). Cells of passages 4 to 6 were used to produce the ECM and to test the ECM effects on osteogenic differentiation.

### 2.4 ECM and single protein substrates deposition

For ECM layer preparation, hiPSC-MPs and BMSCs (pooled from two donors under 30 years for the generation of young adult ECM and from three donors over 70 years for aged ECM) were seeded on 24-well plates (19.000 cells/well) and cultured in 0.5 mL ECM medium consisting of DMEM-HG supplemented with 10% FBS, 100 U/mL P/S, 2 mM L-glutamine, and 50 µM A2P for 10 days. The cultures were kept in a humidified incubator at 37°C and 5% CO2 with media changes twice per week. For control and single protein groups, either standard 24-well tissue culture plates or plates pre-coated for 45 min with single matrix proteins collagen type I (Fisher Scientific, Pittsburgh, United States) or fibronectin (Sigma-Aldrich, St. Louis, United States) at 5 μg/cm^2^ were used.

For iECM deposition on silk fibroin scaffolds, the scaffolds were first pre-coated with 0.1% gelatin and incubated in 500 µL medium for 24 h. The scaffolds were then blot-dried, seeded with 500.000 hiPSC-MPs/scaffold, transferred to 24-well plates, and incubated statically for 1 h. Every 15 min, the scaffolds were flipped upside-down to promote homogenous cell distribution. After seeding, 1 mL of ECM medium was added to each well, and the seeded scaffolds were cultured for 10 days in a humidified incubator at 37°C and 5% CO_2_ with media changes twice per week.

### 2.5 Decellularization

ECM layers and iECM/silk scaffolds were collected, washed with PBS, and decellularized by incubation in 0.5% Triton X-100 buffer containing 20 mM NH_4_OH in PBS for 15 min at 37°C. The treated ECM layers and iECM/silk scaffolds were then washed four times with PBS and incubated in 100 U/mL DNase I solution in DPBS for 1 h. Our procedure was based on the previously published protocols for decellularization of ECM layers on plastic dishes (Sun et al., 2011; Guneta et al., 2018). After the treatment, the ECM layers and iECM/silk scaffolds were washed 3 times with PBS containing P/S (100 U/mL) and stored at 4°C. In order to determine the dry weight, untreated and decellularized scaffolds were lyophilized overnight in ALPHA one to two LSC-BASIC lyophilizer (Martin Christ, Osterode am Harz, Germany).

### 2.6 Osteogenic differentiation of BMSCs on ECM layers, single protein substrates and iECM/silk fibroin scaffolds

BMSCs from three different donors were used in the experiments: a 20-year-old female (F20), a 71-year-old female (F71), and an 89-year-old male (M89). BMSCs were seeded at a density of 5,000/cm^2^ on ECM layers and on tissue culture plastic or single protein substrates in 24-well plates, 0.6 mL of medium was added to each well and they were cultured up to 42 days in either control medium, consisting of DMEM-HG supplemented with 10% FBS, 2 mM L-glutamine and 100 U/mL P/S, or in osteogenic medium consisting of control medium supplemented with 10 nM dexamethasone, 50 µM A2P and 10 mM ß-glycerophosphate, with media changes twice per week.

iECM/silk scaffolds and plain silk scaffolds were incubated in expansion medium 24 h prior to seeding. The scaffolds were then blot-dried, seeded with 500.000 BMSCs on top of each scaffold, and incubated statically for 1 h. Every 15 min, the scaffolds were flipped upside-down to promote homogenous cell distribution in the scaffold. Seeded scaffolds were transferred to fresh 24-well plates, 1 mL of medium was added to each well, and the scaffolds were maintained in static culture in control or osteogenic media for 56 days with media changes thrice per week.

### 2.7 Immunofluorescence staining

Untreated and decellularized ECM layers were fixed with 4% formaldehyde solution in PBS for 15 min at RT, washed, and incubated with 0.1% Triton X-100 in PBS for 5 min. Afterwards, the samples were incubated with primary antibodies against collagen type I (cat. No. ab34710), collagen type IV (cat. No. ab6586), laminin (cat. No. ab11575) and fibronectin (cat. No. ab2413) purchased from Abcam (Cambridge, UK) for 2 h at 4°C in the dark. Primary antibodies were diluted 1:100 in PBS supplemented with 1% (wt/v) BSA. After the incubation, the samples were washed and incubated with a goat anti-rabbit Alexa Fluor^®^ 488 conjugate secondary antibody (cat. No. AP132JA4, Sigma-Aldrich, St. Louis, United States) diluted 1:500 in PBS supplemented with 1% (wt/v) BSA for 1 h at 4°C in the dark. For nuclear visualization, the samples were washed and counterstained with 4′,6-diamidino-2-phenylindole (DAPI) diluted 1:1,000 (wt/v) in PBS for 15 min at 4°C in the dark. After staining, all samples were analyzed on the same day an Axio Observer A1 microscope fitted with ICm1 AxioCam (Carl Zeiss Microscopy, Oberkochen, Germany). Images of untreated and decellularized groups were taken using the same exposure times, which were adapted for each primary antibody and DAPI (taking in account the negative control) as follows: 120 ms collagen type I, 120 ms collagen type IV, 120 ms fibronectin, 180 ms laminin, 60 ms DAPI. Images were overlayed using ImageJ software.

### 2.8 Harvesting of cultured cell-seeded scaffolds

For alkaline phosphatase (ALP) activity assays, calcium assays, and RNA extraction, cultured scaffolds were first cut in half, weighed, and frozen at −80°C. Prior to the analyses, samples were homogenized in appropriate assay buffers using ceramic balls and a Precellys 24 tissue homogenizer (Bertin Technologies SAS, Montigny-le-Bretonneux, France).

### 2.9 DNA content quantification

For DNA content quantification, the samples were incubated in a digestion buffer containing 150 mM NaCl, 55 mM Na Citrate * 2H_2_O, 20 mM EDTA * 2H_2_O, 0.2 M NaH_2_PO_4_ * 1H_2_O, 10 mM EDTA * 2H_2_O, 6 U/mL papain, and 10 mM cysteine in ddH_2_O (with pH 6.0) overnight at 60°C. The digested samples were collected, centrifuged for 5 min at 300 × g, and the DNA content of the supernatant was determined using Hoechst 33,342 dye (Sigma-Aldrich, St. Louis, United States). 100 μL of Hoechst working solution consisting of 5 μg/mL Hoechst dye in assay buffer (2 M NaCl, 50 mM NaH_2_PO_4_, pH 7.4) were added to 50 µL samples or DNA standards of known concentration (calf thymus DNA, Sigma-Aldrich, St. Louis, United States). The samples were incubated for 5 min at 37°C in the dark while slowly shaking, and the fluorescence was measured at 355/460 nm. DNA concentration of the samples was determined using a standard curve constructed with DNA solutions of known concentrations.

### 2.10 ALP activity assay

For the ALP activity assay, the samples were lysed in a solution containing 0.5% Triton X-100 in 0.5 M 2-amino-2-methyl-1-propanol buffer with 2 mM MgCl_2_ (pH 10.3). The lysed samples were centrifuged for 5 min at 300 × g and the ALP activity of the supernatant was determined by adding 50 µL of the pNPP substrate solution (0.02 M) to 100 µL of extracted supernatant and incubation at 37°C. The reaction time until the development of yellow color was recorded, and the reaction was stopped by adding 50 µL of 0.2 M NaOH stop solution. Absorbance was measured for 100 µL sample at 405 nm, and the ALP activity was determined using a standard curve constructed with p-nitrophenol solutions of known concentrations.

### 2.11 Calcium content quantification

For calcium content quantification, samples were extracted in 5% trichloroacetic acid at RT for 30 min. The samples were collected, centrifuged for 10 min at 4°C and the calcium content of the supernatant was determined using calcium (CPC) LiquiColor^®^ test (Stanbio Laboratory, Boerne, United States) according to manufacturer’s instructions.

### 2.12 Gene expression analyses

RNA extraction was performed using the QIAGEN RNeasy Mini Kit (QIAGEN, Hilden, Germany), followed by DNase treatment (Thermo Fisher Scientific, Waltham, United States) according to the manufacturer’s instructions. Approximately 200 ng of extracted RNA was transcribed into cDNA with the GoScript™ Reverse Transcription System 100 (Promega, Wisconsin, United States) using random hexamer primers. Real-time PCR was performed using the CFX96 Real-Time PCR Detection System (Bio-Rad Laboratories, Hercules, United States). 2 μL cDNA were added to a 25 µL volume reaction containing the TaqMan^®^ universal PCR master mix and one of the TaqMan^®^ Gene Expression Assays (Thermo Fisher Scientific, Waltham, United States) for alkaline phosphatase (*ALP,* Hs01029144_m1), osteocalcin (*OCN,* Hs01587814_g1), bone sialoprotein (*BSP*; Hs00173720_m1), and glyceraldehyde 3-phosphate dehydrogenase (*GAPDH*, Hs02786624_g1). Standard cycling conditions were used: 95°C for 10 min, followed by 40 cycles of 95°C for 15 s (denaturation) and 60°C for 60 s (annealing and extension). Results were exported using the CFX Manager 3.1 (Bio-Rad Laboratories, California, United States) and analyzed in Excel (Microsoft, Redmond, United States) using the ∆∆Ct method. Expression levels of the target osteogenic genes were normalized to the expression level of the housekeeping gene *GAPDH*.

### 2.13 Histological stainings of 2D cultures

Upon BMSC differentiation, the samples were washed with PBS and fixed with 4% formaldehyde in PBS for 10 min at RT. All stainings were documented using an inverted microscope fitted with color camera (Zeiss Primovert Microscope with ICc5 AxioCam).

Alizarin Red staining was used to visualize calcium deposition. The samples were washed with distilled water and incubated in 2% Alizarin Red S solution (pH 4.2) for 30 min. Afterwards, Alizarin Red S solution was removed, the samples were extensively rinsed with distilled water, and analyzed.

Collagen deposition was visualized using Picrosirius Red staining. Samples were incubated in Weigert’s haematoxylin for 5 min, then shortly treated with 0.25% HCl and afterwards washed for 10 min with tap water. The samples were then incubated in a 0.1% (wt/v) Sirius red F3B (Direct Red 80) (Sigma-Aldrich, St. Louis, United States) in saturated picric acid solution for 1 h at RT, followed by rinsing with 0.5% acetic acid. Histological stainings were semi quantitatively analyzed using ImageJ software (National Institutes of Health, Bethesda, United States). Four to six non-overlapping images were taken for analysis in each group. Thresholding was performed by catching the same transition for each picture using histograms. The area stained positive was measured for each sample and calculated against negative control.

Adipogenic differentiation was visualized using Oil Red O staining. 3 g/L Oil Red O stock solution was prepared in 2-propanol. Samples were first washed with distilled water and afterwards with 70% EtOH. Then, the samples were incubated in Oil Red O working solution (prepared by mixing the stock solution with ddH_2_O at 3:2) for 15 min at RT. The Oil Red O working solution was removed, samples were washed with distilled water, and analyzed.

### 2.14 Histological/immunohistochemical analyses of 3D cultures

For histological evaluations, the cultured 3D constructs were fixed in 4% formaldehyde in PBS for 24 h at RT, followed by dehydration in graded ethanol solutions and embedding in paraffin. Afterwards, the fixed construct samples were sectioned into 4 µm thin sections and kept in a 37°C incubator. Prior to all stainings, sections were deparaffinized and rehydrated.

For an overview of the tissue morphology, haematoxylin and eosin (H&E) staining was performed following a standard protocol. In order to detect tissue mineralization, sections were stained with 0.5% aqueous silver nitrate solution (von Kossa staining). Immunohistochemical stainings were conducted using primary antibodies against collagen type I (dilution 1:100 (v/v), cat. Number PA1-26204, Thermo Fisher Scientific, Waltham, United States), collagen type IV (dilution 1:700 (v/v), cat. No. Ab19808, Abcam, Cambridge, UK), fibronectin (dilution 1:100 (v/v), cat. No. MA5-11981, Thermo Fisher Scientific, Waltham, United States), laminin 5 (dilution 1:50 (v/v), cat. No. AB19562, Merck KGaA, Darmstadt, Germany), and OCN (dilution 1:200 (v/v), cat. No. AB10911, Merck KGaA, Darmstadt, Germany). Epitopes were retrieved as follows: for collagen type I sections were treated with pepsin (10 min at 37°C), for collagen type IV sections were steamed in 10 mM EDTA buffer (pH 9) for 20 min, for osteocalcin sections were steamed in 10 mM sodium citrate buffer (pH 6) for 20 min, and for laminin 5 and fibronectin sections were incubated with Proteinase K (Agilent Technologies, Santa Clara, United States) for 8 min at RT. Following a blocking step using Bloxall^®^ (SP6000, Vector, Burlingame, United States) for 10 min at RT, sections were incubated with primary antibodies for 1 h at RT and then rinsed three times with TBS buffer. Secondary horseradish peroxidase (HRP) conjugated antibodies obtained from ImmunoLogic (cat. No. VWRKDPVM110HRP or VWRKDPVR110HRP, Duiven, Netherlands) were incubated on the sections for 30 min at RT. After a final rinsing step, the detection was done with ImmPACTTMNova RedTM (SK4805, Vector, Burlingame, United States) followed by a Haemalaun counterstaining. The stainings were documented using light microscopy (Axioplan2 imaging, Carl Zeiss Microscopy, Oberkochen, Germany and Olympus BX61VS., Olympus Corporation, Tokyo, Japan).

### 2.15 Statistical analyses

Decellularization experiments were performed four times for iECM layer and three times for 3D iECM/silk scaffolds. Effects of iECM layer on BMSC growth and osteogenesis were tested in two independent experiments, once with BMSCs from three donors separately and once with BMSCs from donor M89, always including cell culture plastic as controls. Effects of iECM/silk scaffolds on BMSC growth and osteogenesis were tested in three independent experiments, once with BMSCs from two donors (F20 and M89) and twice with BMSCs from donor M89. Comparison of iECM with BMSC-ECM and cell culture plastic control groups was performed once with BMSCs from donor M89 and cells pooled from two young adult and from three aged donors to generate the yECM and aECM with minimal inter-donor variation. Comparison of iECM with single ECM protein substrates was conducted once using two protein substrates separately and BMSCs from donor M89.

All data are presented as mean ± standard deviation (SD). Normal data distribution was evaluated, and the differences between the groups were evaluated accordingly using unpaired *t*-test, one-way Kruskal–Wallis test with Dunn’s multiple comparison test, or ANOVA with Tukey’s multiple comparison test with a *p*-value of 0.05 considered as statistically significant. All analyses were performed using GraphPad Prism 9 software (GraphPad Software, San Diego, United States).

## 3 Results

### 3.1 iECM engineered from hiPSC-MPs in 2D and 3D culture retains structural proteins after decellularization, while remaining nearly DNA-free

**FIGURE 1:**
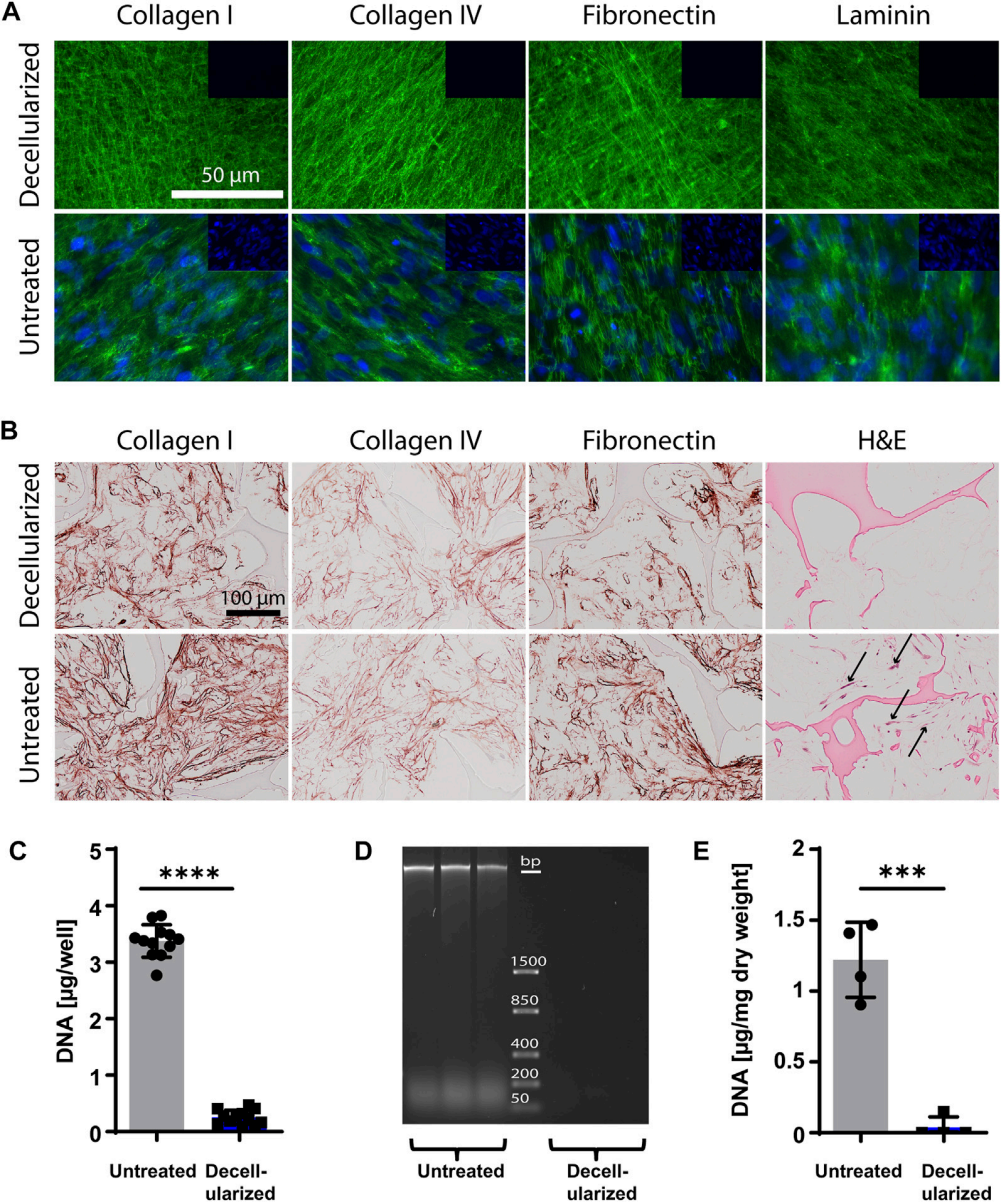
Characterization of iECM layers and 3D iECM/silk scaffold constructs before and after decellularization. **(A)** Decellularized and untreated iECM layers stained positive for collagen type I, collagen type IV, fibronectin and laminin (green). Cell nuclei (blue) were present only in untreated iECM layers. Insets (top right of each picture) show negative staining controls. Scale bar: 100 µm. **(B)** Untreated and decellularized iECM/silk constructs stained positive for collagen type I, collagen type IV and fibronectin. Haematoxylin and eosin (H&E) staining indicated successful cell removal after decellularization. Black arrows indicate cells. Scale bar: 100 µm. **(C)** DNA content evaluation of iECM layers confirmed efficient DNA removal. Data represents mean ± SD (*n* = 12). **(D)** Gel electrophoresis showed the absence of residual DNA in decellularized iECM/silk constructs (*n* = 3). **(E)** DNA content evaluation of iECM/silk constructs confirmed efficient DNA removal. Data represents mean ± SD (*n* = 4). **(C and E)** Statistically-significant differences between the groups were evaluated using unpaired *t*-test and are marked with: ***p < 0.001; ****p < 0.0001.

HiPSC-MPs were cultured on standard tissue culture dishes (2D) and in 3D silk scaffolds for 10 days, and the deposited iECM was examined before and after the decellularization procedure by immunofluorescent and immunohistochemical stainings as well as DNA content quantification (Figure 1). The untreated iECM layer on plastic dishes stained positive for collagen type I, collagen type IV, fibronectin and laminin, as well as nuclear DNA. Following decellularization, the staining for structural proteins in the iECM layer remained strongly positive. In contrast, a nuclear DNA signal was not detected, suggesting an efficient removal of cellular material (Figure 1A). Similarly, 3D iECM/silk constructs stained positive for collagen type I, collagen type IV, and fibronectin before and after decellularization, whereas the cells were removed after decellularization, as observed after H&E staining (Figure 1B). Effective decellularization of iECM layer was confirmed by DNA content quantification, which showed a significant reduction after decellularization, with 0.25 μg DNA/well remaining (Figure 1C). Effective decellularization of iECM/silk constructs was confirmed by gel electrophoresis, which showed no residual DNA in decellularized samples (Figure 1D) and by DNA content quantification, which showed a significant decline in the DNA content, with 37 ng DNA/mg dry weight remaining in decellularized iECM/silk scaffolds (Figure 1E).

### 3.2 iECM enhances adult/aged human BMSC proliferation in 2D culture

**FIGURE 2:**
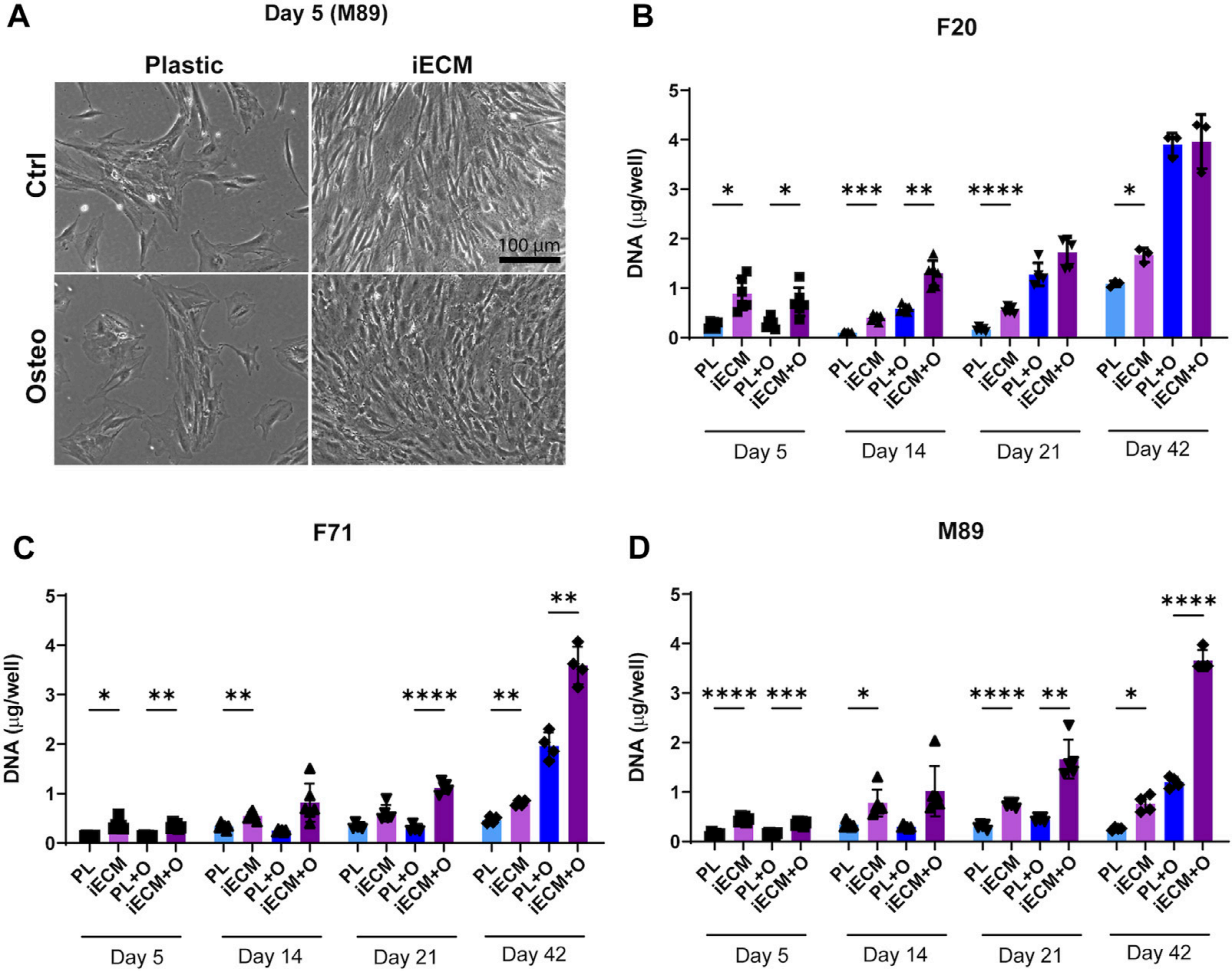
Enhanced proliferation of human BMSCs on iECM layer. **(A)** Representative images of enhanced cell growth (donor M89) after 5 days of culture on the iECM layer as compared to standard tissue culture plastic in control and osteogenic media. Scale bar: 100 µm. Group labels: Ctrl—control medium, Osteo—osteogenic medium. **(B–D)** DNA content quantification of F20 **(B)**, F71 **(C)** and M89 cultures **(D)** on the iECM layer and tissue culture plastic. Group labels: PL—plastic with control medium, iECM—iECM layer with control medium, PL + O- plastic with osteogenic medium, iECM + O- iECM layer with osteogenic medium. Data represents mean ± SD (*n* = 4). Statistically-significant differences between the groups were evaluated using two-way ANOVA, followed by Tukey’s multiple comparison test, and are marked with: *p < 0.05; **p < 0.01; ***p < 0.001; ****p < 0.0001.

Human BMSCs from one young adult and two aged donors were grown on an iECM layer and on tissue culture plastic in control or osteogenic media for up to 42 days (Figure 2). As early as 5 days after seeding, a striking difference in cell proliferation could be observed in which cells in both culture media covered a large portion of the iECM layer, but not of the tissue culture plastic (Figure 2A; Supplementary Figure S1). DNA content quantification indicated a significantly higher proliferation of the young 20-year-old female BMSCs in control medium on the iECM layer as compared to tissue culture plastic at all time points (Figure 2B). In osteogenic medium, DNA content of F20 cells on the iECM layer was significantly higher as compared to tissue culture plastic after 5 and 14 days, whereas after 21 and 42 days, no significant difference remained (Figure 2B). In the aged 71-year-old female BMSCs (Figure 2C) and 89-year-old male BMSCs (Figure 2D), DNA content was significantly higher on the iECM layer as compared to plastic control in both culture media after 5 days and 42 days. After 14 days and 21 days of culture, a significantly higher DNA content, or a trend to higher DNA content was observed on the iECM as compared to the tissue culture plastic for BMSCs of both donors in both culture media. Taken together, our DNA content quantification suggests that culture on the iECM layer enhances young adult and aged human BMSC proliferation.

### 3.3 iECM enhances adult/aged human BMSC osteogenic differentiation in 2D culture

**FIGURE 3:**
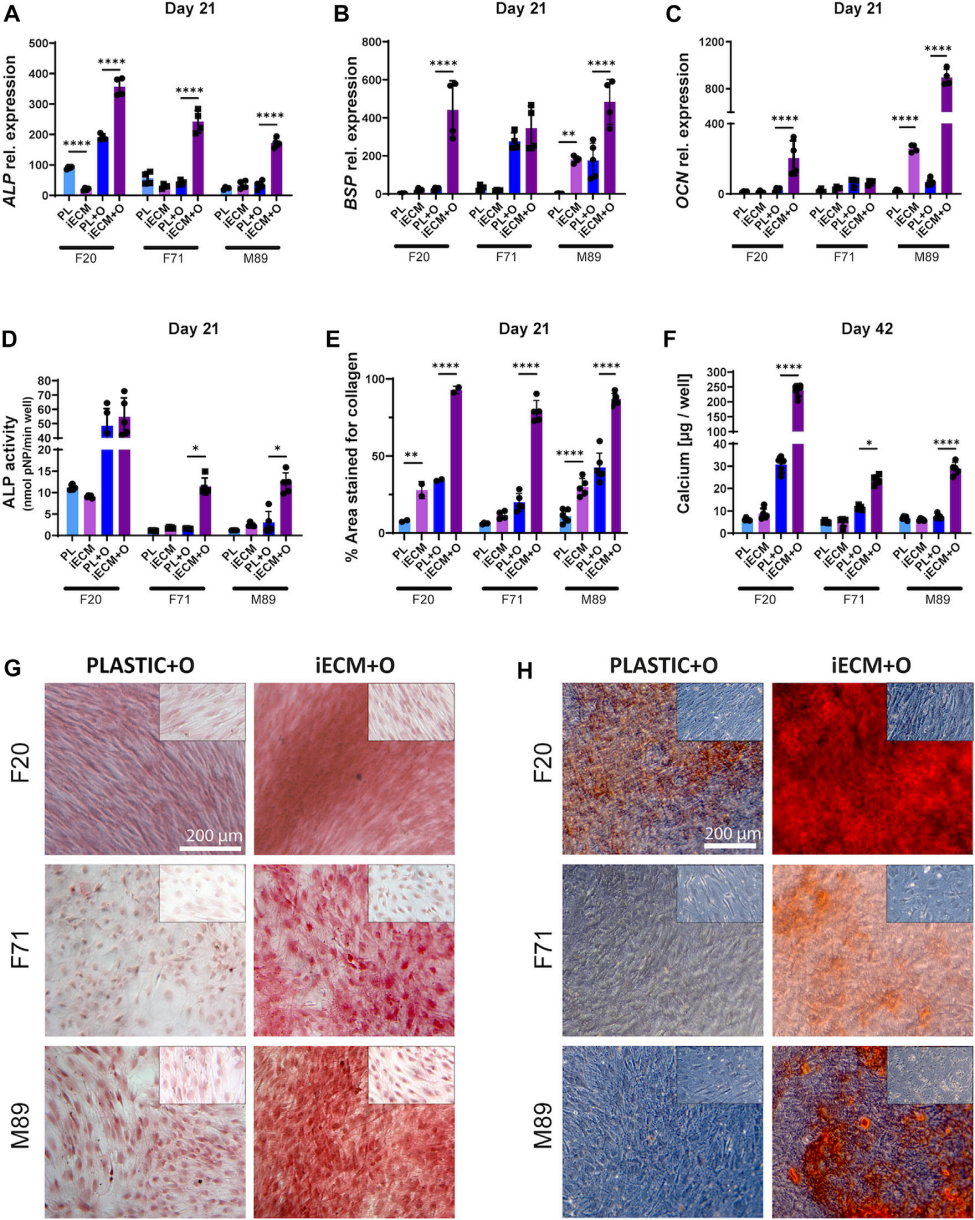
Enhanced osteogenic differentiation of human BMSCs grown on iECM layer. (A–C) Relative gene expression levels of osteogenic markers alkaline phosphatase (*ALP*, **(A)**, bone sialoprotein (*BSP*, **(B)** and osteocalcin (*OCN*, **(C)** after 21 days of culture. **(D)** ALP activity after 21 days of culture. **(E)** Percentage of culture area stained positive for collagen deposition after 21 days of culture. **(F)** Calcium content quantification after 42 days of culture. **(A–F)** Group labels: PL—plastic with control medium, iECM—iECM layer with control medium, PL + O- plastic with osteogenic medium, iECM + O–iECM layer with osteogenic medium. Data represents mean ± SD (*n* = 4). Statistically-significant differences between the groups were evaluated using two-way ANOVA, followed by Tukey’s multiple comparison test, and are marked with: *p < 0.05; **p < 0.01; ****p < 0.0001. **(G)** Picrosirius Red staining of collagen deposition after 21 days of culture. **(H)** Alizarin Red staining for mineral deposition after 42 days of culture. **(G,H)** Insets (top right of each picture) show MSC cultures in control medium for iECM and tissue culture plastic groups. Group labels: Plastic + O- plastic with osteogenic medium, iECM + O- iECM with osteogenic medium. Scale bars: 200 µm.

We next evaluated osteogenic differentiation of the BMSCs from three donors growing on an iECM layer or on tissue culture plastic in control and osteogenic media for up to 42 days (Figure 3). Gene expression analysis showed a significant upregulation of *ALP* gene expression in BMSCs of all three donors after 21 days of culture in osteogenic medium on the iECM as compared to tissue culture plastic (Figure 3A). Interestingly, F20 cells cultured in control medium had a significantly lower *ALP* gene expression on the iECM as compared to tissue culture plastic at this timepoint. Furthermore, gene expression of late osteogenic markers *BSP* and *OCN* was significantly upregulated in the F20 cells cultured in osteogenic medium and in the M89 cells cultured in either medium on iECM as compared to tissue culture plastic (Figures 3B,C). No statistically significant differences in *BSP* and *OCN* expression between iECM and tissue culture plastic could be observed in the F71 cells.

Analysis of ALP enzyme activity after 21 days of culture exhibited a pattern similar to the *ALP* gene expression, with significantly higher activity in F71 and M89 cells cultured in osteogenic medium on the iECM as compared to tissue culture plastic (Figure 3D). Overall, the ALP activity determined in F20 BMSCs was higher compared to the aged donor BMSCs. However, there was no significant difference in the ALP activity of F20 cells between the iECM and tissue culture plastic in either culture media.

In order to characterize the deposition of collagen matrix, histochemical staining with Picrosirius Red was performed and quantified (Figures 3E,G). After 21 days of culture, a significantly higher area (around 80% on average) stained positive for collagen in BMSC cultures in osteogenic medium on the iECM. In osteogenic BMSC cultures on tissue culture plastic, only around 30% of the area was stained positive (Figure 3E). A similar effect, as in a larger area staining positive for collagen on the iECM as compared to tissue culture plastic, was observed in control medium. Moreover, we found a significant increase in calcium content for BMSCs of all three donors cultured in osteogenic medium on the iECM as compared to tissue culture plastic (Figure 3F). The increase in calcium content corresponded with increased mineralization determined by Alizarin Red staining (Figure 3H). In F20 BMSCs, Alizarin Red staining showed calcium deposition by cells cultured on tissue culture plastic in osteogenic medium but not in control medium. Notably, F20 cells grown on iECM in osteogenic medium exhibited an increased calcium deposition as compared to tissue culture plastic (Figure 3H). In contrast, aged BMSCs F71 and M89 failed to deposit calcium in osteogenic medium on tissue culture plastic, but exhibited calcium deposition in osteogenic cultures on iECM.

### 3.4 iECM enhances adult/aged human BMSC osteogenic differentiation in 3D culture

**FIGURE 4:**
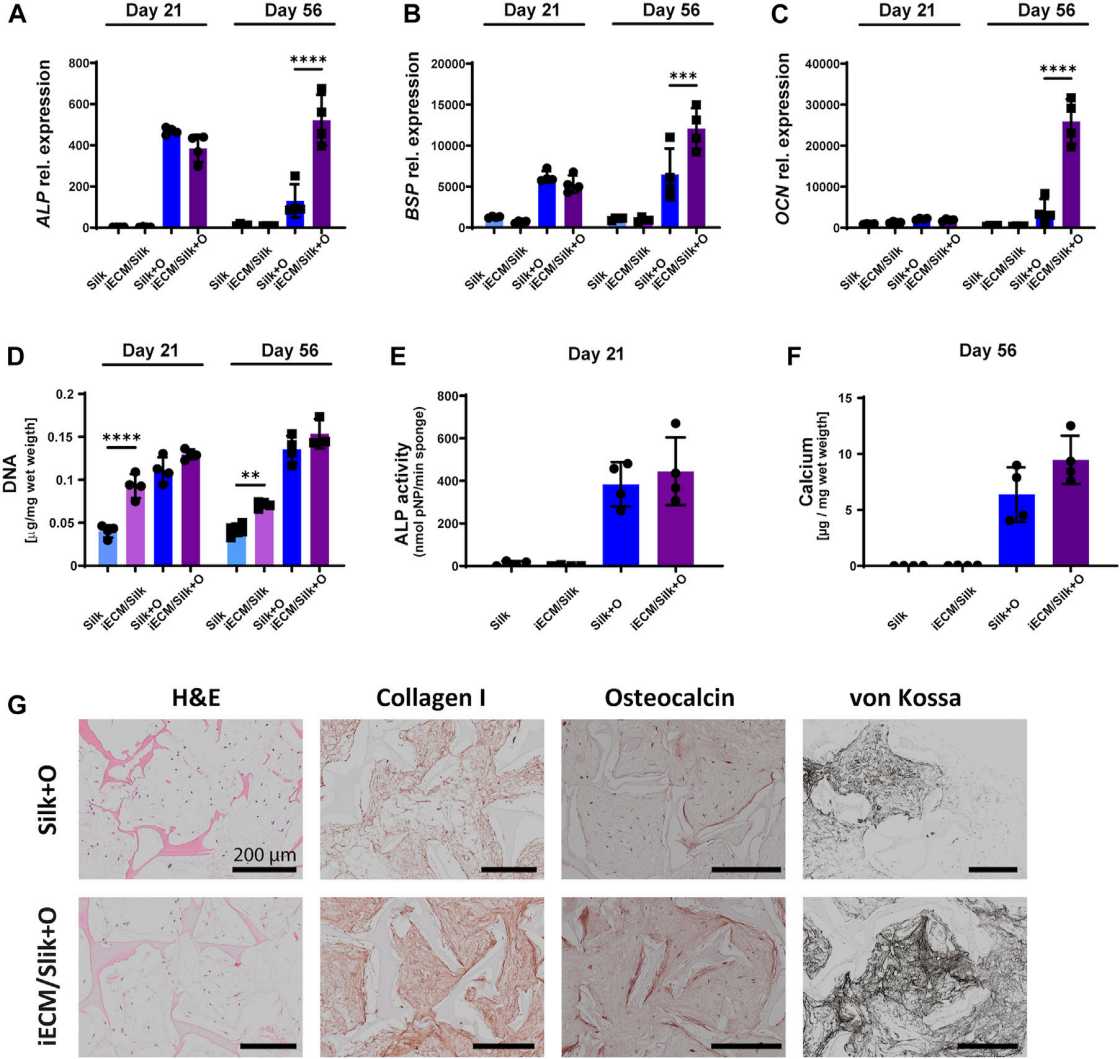
Enhanced osteogenic differentiation of aged human BMSCs grown in 3D iECM/silk scaffolds. **(A–C)** Relative gene expression levels of osteogenic markers alkaline phosphatase (*ALP*, **(A)**, bone sialoprotein (*BSP*, **(B)** and osteocalcin (*OCN*, **(C)** after 21 and 56 days of culture. **(D)** DNA content quantification after 21 and 56 days of culture. **(E)** ALP activity after 21 days of culture. **(F)** Calcium content quantification after 56 days of culture. **(A–F)** Group labels: Silk—plain silk scaffold with control medium, iECM/silk–iECM/silk scaffold with control medium, Silk + O- plain silk scaffold with osteogenic medium, iECM/silk + O–iECM/silk scaffold with osteogenic medium. Data represents mean ± SD (*n* = 4). Statistically significant differences between the groups were evaluated using a Kruskal–Wallis test, followed by Dunn’s multiple comparison test **(E and F)**, and two-way ANOVA followed by Tukey’s multiple comparison test **(A–D)**, and are marked with: **p < 0.01; ***p < 0.001; ****p < 0.0001. **(G)** Histological/immunohistochemical analyses of BMSCs cultured on plain silk scaffolds and iECM/silk scaffolds after 56 days of culture. Group labels: Silk + O- plain silk scaffold with osteogenic medium, iECM/silk + O- iECM/silk scaffold with osteogenic medium. Scale bars represent 200 µm.

After we demonstrated an improvement of young adult- and aged BMSC proliferation and osteogenic differentiation on iECM in 2D culture, we evaluated whether its stimulatory effect on BMSCs could be achieved in a 3D culture environment. BMSCs of donors F20 and M89 were seeded in iECM/silk scaffolds and in plain silk scaffolds and cultured in control and osteogenic media for up to 56 days (Figure 4; Supplementary Figure S2). Gene expression analysis showed no significant differences in *ALP*, *BSP,* and *OCN* expression in aged M89 BMSCs between iECM/silk scaffolds and plain silk scaffolds after 21 days of culture in either culture media (Figures 4A–C). However, after 56 days of culture in osteogenic medium, a significantly higher expression of all three osteogenic markers was observed in M89 cells in iECM/silk scaffolds as compared to plain silk scaffolds. Similarly, a trend or a significantly higher expression of the three osteogenic markers was observed for in F20 cells in iECM/silk scaffolds as compared to plain silk scaffolds cultured in osteogenic medium for 56 days (Supplementary Figure S2).

Furthermore, DNA content quantification indicated a significantly higher M89 BMSCs proliferation on iECM/silk scaffolds as compared to plain silk scaffolds after 21 and 56 days of culture when control medium was used (Figure 4D). DNA content of the scaffolds cultured in osteogenic medium was overall higher than in control medium, with iECM/silk scaffolds exhibiting a trend towards higher DNA quantities as compared to plain silk scaffolds after 21 and 56 days. For F20 BMSCs, DNA content quantification indicated a comparable proliferation in both scaffold groups cultured in control medium and a significantly lower proliferation on iECM/silk scaffolds as compared to plain silk scaffolds cultured in osteogenic medium (Supplementary Figure S2).

M89 BMSCs ALP activity after 21 days of culture (Figure 4E) and calcium content after 56 days of culture (Figure 4F) trended upwards in the iECM/silk scaffolds compared with plain silk scaffolds, and F20 BMSCs exhibited a significantly higher calcium content after 56 days of culture in the iECM/silk scaffolds compared with plain silk scaffolds (Supplementary Figure S2). Histological analyses performed after 56 days demonstrated an increased deposition of collagen type I, OCN, and mineral by BMSCs cultured in osteogenic medium on iECM/silk scaffolds as compared to plain silk scaffolds (Figure 4G; Supplementary Figure S2). Taken together, our data suggest a positive effect of iECM deposited on silk scaffolds on osteogenesis of young adult and aged BMSCs cultured *in vitro*.

### 3.5 iECM and BMSC-ECM similarly enhance the growth and osteogenic differentiation of aged human BMSCs

**FIGURE 5:**
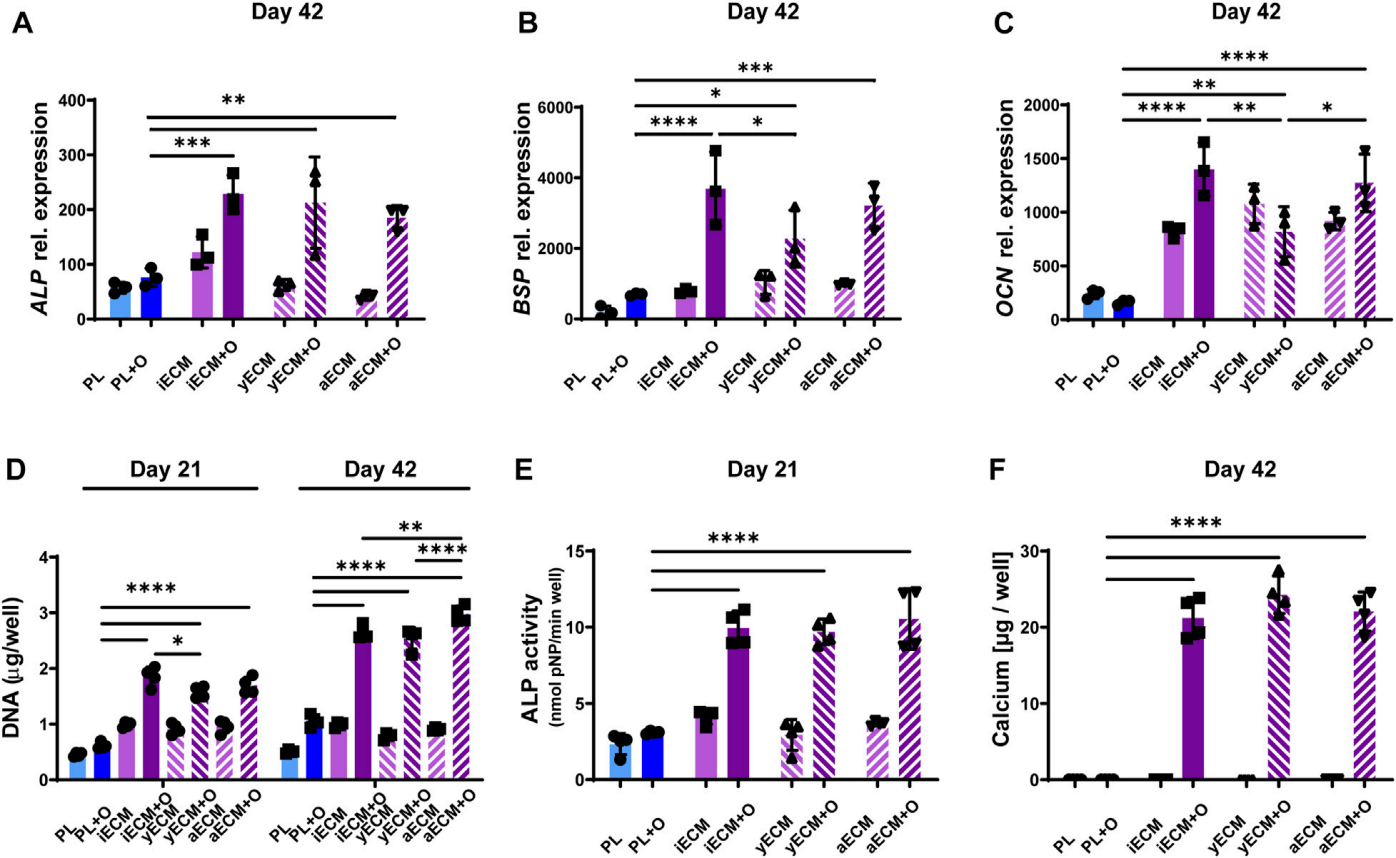
Enhanced osteogenic differentiation of aged human BMSCs grown on ECM layers derived from cells of different origins. **(A–C)** Relative gene expression levels of osteogenic markers alkaline phosphatase (ALP, **(A)**, osteopontin (BSP, **(B)** and osteocalcin (OCN, **(C)** after 42 days of culture. **(D)** DNA content quantification after 21 and 42 days of culture. **(E)** ALP activity after 21 days of culture. **(F)** Calcium content quantification after 42 days of culture. **(A–F)** Group labels: PL–plastic control with control medium, iECM - iECM layer with control medium, yECM—young adult BMSC-ECM layer with control medium, aECM - aged BMSC-ECM layer with control medium, PL + O- plastic control with osteogenic medium, iECM + O- iECM layer with osteogenic medium, yECM + O- young adult BMSC-ECM layer with osteogenic medium, aECM + O- aged BMSC-ECM layer with osteogenic medium. Data represents mean ± SD (*n* = 4). Statistically-significant differences between the groups were evaluated using Kruskal–Wallis test, followed by Dunn’s multiple comparison test **(A,B,C,E and F)**, and two-way ANOVA, followed by Tukey’s multiple comparison test **(D)**, and are marked with: *p < 0.05; **p < 0.01; ***p < 0.001; ****p < 0.0001.

We next compared the effects of iECM to the effects of ECMs generated from young adult BMSCs (yECM) and aged BMSCs (aECM) using the same procedure (Supplementary Figure S3). M89 BMSCs were cultured on the three ECMs and on tissue culture plastic in control and osteogenic media for up to 42 days (Figure 5). Gene expression analysis showed a similar upregulation of *ALP* expression in M89 cells grown on all three ECMs as compared to tissue culture plastic after 42 days of culture in osteogenic medium (Figure 5A). Gene expression of late osteogenic markers *BSP* and *OCN* was also significantly upregulated when cells were grown on any of the three ECMs as compared to tissue culture plastic after 42 days of culture in osteogenic medium (Figures 5B,C). Furthermore, differences were found between cells grown on different ECMs in osteogenic medium. *BSP* expression was significantly higher when grown on iECM as compared to yECM, whereas *OCN* expression was significantly lower when grown on yECM as compared to iECM and aECM.

DNA content quantification indicated a significantly higher M89 cell proliferation in osteogenic medium when grown on either of the three ECMs as compared to tissue culture plastic after 21 and 42 days (Figure 5D). Interestingly, comparison between the three ECMs after 42 days showed that DNA content was also significantly higher in osteogenic medium on aECM as compared to iECM and yECM.

ALP enzyme activity of M89 cells after 21 days of culture (Figure 5E) exhibited a pattern similar to that of *ALP* gene expression (Figure 5A), with significantly higher activity in M89 cells cultured in osteogenic medium on any of the three ECMs as compared to tissue culture plastic. Moreover, we found a significant increase in calcium content when M89 cells were cultured in osteogenic medium on any of the three ECMs as compared to tissue culture plastic (Figure 5F). No calcium deposition could be detected in the control medium groups. Taken together, these data show a comparable stimulatory effect of iECM and BMSC-ECM on BMSC proliferation and osteogenesis, and the BMSC age and cellular reprogramming origin of the hiPSC-MPs appear to have no negative impact.

### 3.6 iECM enhances osteogenic differentiation of aged human BMSCs more than single protein substrates

**FIGURE 6:**
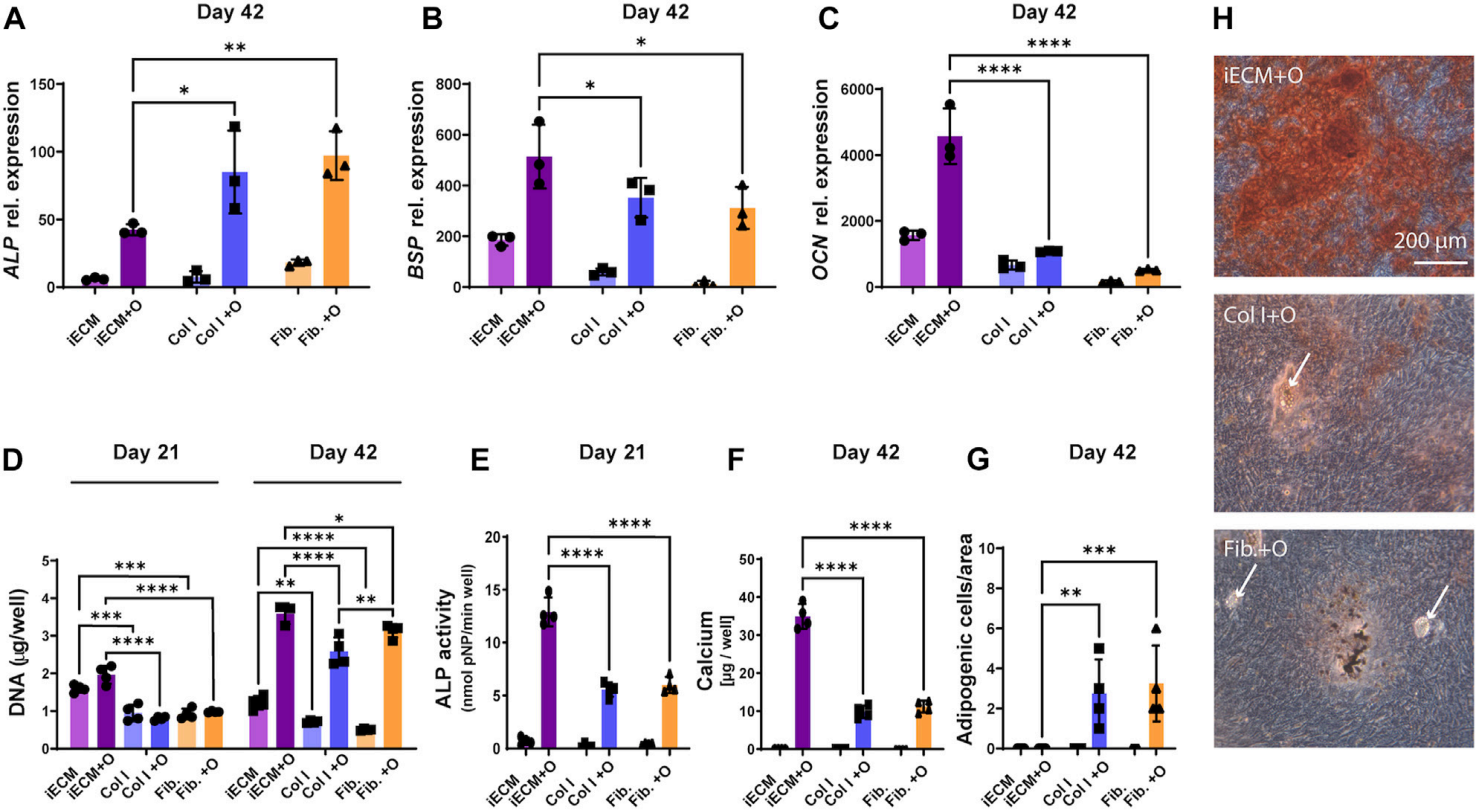
Enhanced osteogenic differentiation of aged human BMSCs grown on iECM layer compared with single protein substrates. **(A–C)** Relative gene expression levels of osteogenic markers alkaline phosphatase (ALP, **(A)**, bone sialoprotein (BSP, **(B)** and osteocalcin (OCN, **(C)** after 42 days of culture. **(D)** DNA content quantification after 21 and 42 days of culture. **(E)** ALP activity after 21 days of culture. **(F)** Calcium deposition after 42 days of culture. **(G)** Adipogenic cell quantification after 42 days of culture. **(A–G)** Group labels: iECM—iECM layer with control medium, Col I—collagen I with control medium, Fib—fibronectin with control medium, iECM + O- iECM layer with osteogenic medium, Col I + O- collagen I with osteogenic medium, Fib + O- fibronectin with osteogenic medium. Data represents mean ± SD (*n* = 4). Statistically-significant differences between the groups were evaluated using Kruskal–Wallis, test followed by Dunn’s multiple comparison test **(A,B,C,E,F and G)**, and two-way ANOVA, followed by Tukey’s multiple comparison test **(D)**, and are marked with: *p < 0.05; **p < 0.01; ***p < 0.001; ****p < 0.0001. **(H)** Alizarin Red staining for mineral deposition after 42 days of culture. Group labels: iECM + O- iECM layer with osteogenic medium, Col I + O- collagen I with osteogenic medium, Fib + O- fibronectin with osteogenic medium. Scale bars represent 200 µm. White arrows indicate adipocytes.

After establishing that the iECM enhances the osteogenic capacity of BMSCs in 2D and 3D culture, we wanted to determine whether single ECM protein substrates could have a similarly high stimulatory effect as the iECM (Figure 6). Interestingly, M89 cells exhibited a significant upregulation of *ALP* gene expression when cultured on collagen or fibronectin substrates in osteogenic medium for 42 days as compared to the iECM (Figure 6A). However, gene expression of late osteogenic markers *BSP* and *OCN* was significantly higher when cells were grown on the iECM as compared to single protein substrates after 42 days of culture in osteogenic medium (Figures 6B,C).

DNA content quantification indicated significantly higher M89 cell proliferation on the iECM layer as compared to single protein substrates for both culture media after 21 and 42 days (Figure 6D). Interestingly, after 42 days, we also observed a significantly higher DNA content on fibronectin as compared to collagen under osteogenic conditions.

In contrast to *ALP* gene expression, ALP enzyme activity of M89 cells in osteogenic medium after 21 days was significantly higher on the iECM as compared to single protein substrates (Figure 6E). This increase was reflected by the significant increase in calcium content of M89 cultures in osteogenic medium on the iECM for 42 days as compared to single protein substrates (Figure 6F). Increased calcium content corresponded with increased mineralization of cells cultured on iECM as compared to single protein substrates (Figure 6H). Interestingly, the increase in aged BMSC osteogenic differentiation coincided with a decrease in formation of adipogenic cells under osteogenic conditions (Figures 6G,H). No adipogenic cells could be found on the iECM, whereas a significant number of adipogenic cells could be observed when M89 cells were cultured in osteogenic medium on single protein substrates (Figure 6G). Taken together, these data suggest a stronger stimulatory effect of the iECM on aged BMSC osteogenesis compared to selected single proteins, with an accompanying inhibitory effect on adipocyte formation.

## 4 Discussion

HiPSCs represent a unique source of rejuvenated human progenitor cells, as well as a production system for bioactive human tissue components that can be standardized and scaled-up for clinical translation. In the current study, we investigated the potential of iECM derived from hiPSC-MPs to promote the bone forming potential of primary human BMSCs either as a layer in 2D tissue culture dishes or as a 3D silk-fibroin scaffold coating. We found that iECM strongly enhanced the osteogenesis of young adult BMSCs, as well as partially restored the impaired osteogenesis of aged BMSCs. We further found that the osteogenic capacity enhancement was comparable between the iECM engineered from hiPSC-MPs and the ECMs from primary BMSCs of young adult- and aged donors. However, iECM had a stronger stimulatory effect on aged BMSC osteogenesis compared with two single matrix protein coatings, and it also decreased the formation of adipocytes from aged BMSCs in osteogenic conditions.

Native ECM of mesenchymal tissues is a complex environment that regulates processes of tissue development and repair (Clark and Keating, 1995; Hocking et al., 1998). *In vitro* ECM engineering is being studied as an alternative to native tissue-derived ECM, as it offers a chance to modulate the ECM properties and combine it into hybrid biomaterials (Zhang et al., 2016). For the repair of bone and cartilage defects, ECM engineering can be used to provide inert synthetic and natural scaffolds a bioactive coating, promoting their integration and tissue regeneration by endogenous cells. ECM was previously generated from primary MSCS of different tissue origins (Sadr et al., 2012; Antebi et al., 2015; Silva et al., 2020; Liao et al., 2010; Datta et al., 2005). However, primary MSCs present significant challenges for scale-up and standardization. In contrast, hiPSCs present a scalable source of rejuvenated human mesenchymal progenitors and tissue components (Wiegand and Banerjee, 2019; de Peppo and Marolt, 2013). For clinical translation, a common, fully-characterized hiPSC cell line could be used for ECM engineering (Abraham et al., 2017), followed by an efficient removal of allogeneic ECM-producing cells in the process of decellularization. Additional safety could be provided by using hypo-immune hiPSC lines that exhibit a restricted immune response due to B2M gene knockout (Chen et al., 2023).

In order to avoid unwanted immune responses to foreign cells by engineered ECM (Manfredi et al., 2009; Zhang et al., 2010; Gall et al., 2012), minimal decellularization requirements have previously been defined as: i) less than 50 ng double stranded DNA per mg dry weight of ECM; ii) residual DNA fragments shorter than 200 bp; and iii) lack of visible nuclear material in tissue sections stained with DAPI or H&E (Crapo et al., 2011). In our study, these requirements were fulfilled after the iECM decellularization using a combination of Triton X-100/NH_4_OH and DNase treatment (Zhang et al., 2016; Lin et al., 2012; Lai et al., 2013; Sun et al., 2011). Upon decellularization, staining for collagen type I, collagen type IV, fibronectin, and laminin in the iECM layer remained positive. However, the effect of Triton X-100 on mechanical properties and bioactivity of iECM by selective detergent solubilization of non-collagenous components cannot be excluded (Cartmell and Dunn, 2000; Reing et al., 2010). In 3D iECM/silk scaffolds, immunohistological staining confirmed the deposition and retention of iECM containing collagen type I, collagen type IV, and fibronectin after decellularization.

Our decellularized iECM strongly enhanced the proliferation of both young adult and aged primary human BMSCs in 2D culture. This finding is in line with prior studies demonstrating that human MSC-derived ECMs promote young adult MSC proliferation as compared with standard tissue culture plastic (Lai et al., 2010; Ng et al., 2014; Chen et al., 2007; Yang et al., 2018). In fact, it was previously shown that ECMs derived from different MSC sources and neonatal fibroblasts share a common set of proteins, whereas the cell-specific, unique matrisome signatures of the individual ECMs had a minimal impact on MSC growth (Ragelle et al., 2017; Prewitz et al., 2013).

We next evaluated the effects of the iECM layer on BMSC osteogenesis using a combination of early osteogenic marker ALP, late osteogenic markers BSP and OCN, and collagen matrix deposition and mineralization (Malaval et al., 1994; Liu et al., 1994). In agreement with prior studies (Pham et al., 2008), we found an overall enhancement of osteogenesis in both young adult and aged BMSCs cultured on the iECM layer, with some specific differences between the donors. In particular, the ALP enzyme activity level was higher in young adult BMSCs compared with aged BMSCs, but it was not significantly different between the iECM layer and tissue culture plastic in young adult BMSCs. In contrast, ALP activity was significantly increased for BMSCs from both aged donors on the iECM. Gene expression levels of the three osteogenic markers were significantly increased in young adult BMSCs and aged BMSCs of one donor, whereas BMSCs of the other aged donor only exhibited increased *ALP* gene expression on the iECM. These differences are most likely due to the individual BMSC strain variation, such as differential expression of integrin and growth factor receptors on the cell surface (Nieto-Nicolau et al., 2020; Samsonraj et al., 2017)

Collagen type I is one of the major organic components of the mineralized bone matrix (Oosterlaken et al., 2021). It has been shown that an increased collagen matrix deposition leads to an increased calcium deposition (Lynch et al., 1995; Kihara et al., 2006; Chiu et al., 2012). A concurrence of increased collagen matrix and calcium deposition was found for BMSCs of all three donors differentiating on iECM in our study. However, similar to ALP activity, the extent of calcified matrix deposition by aged BMSCs did not quantitatively match the level of calcified matrix deposition found with young adult BMSCs. Additional strategies such as elimination of senescent, non-functional cells from the population could be employed in conjunction with iECM engineering to further enhance the overall osteogenic response (Farr and Khosla, 2019; Wang et al., 2021).

The majority of prior studies tested the osteoinductive actions of engineered ECMs with young adult MSCs, which do not reflect the impaired bone formation potential of aged cells (Infante and Rodríguez, 2018). We therefore used BMSCs of young adult and one of the two aged donors to evaluate the iECM effect on osteogenesis in a 3D scaffold environment. We observed enhanced proliferation of aged BMSCs on the iECM/silk scaffolds in control medium, whereas in osteogenic medium, the cell proliferation was higher overall in both scaffold groups compared to control medium, with no significant difference between the scaffold groups. DNA content levels were higher in young adult BMSCs compared with aged BMSCs. For young adult BMSCs, there were no significant differences between the two scaffold groups when cultured in control medium, whereas when cultured in osteogenic medium, DNA content indicated slightly lower proliferation on the iECM/silk scaffolds as compared to plain silk scaffolds. These findings might be partially attributed to cell age, to cell growth being limited by the available pore spaces in iECM/silk scaffolds as compared to plain silk scaffolds during the 56 days culture period, and to enhancement of MSC proliferation by the osteogenic medium supplements (Jaiswal et al., 1997). Furthermore, the 3D iECM/silk enviroment resulted in increased gene expression of osteogenic markers as compared to plain silk scaffolds in BMSCs of both donors, and a denser deposition of mineralized bone-like ECM containing collagen type I and OCN. These findings are in line with previous studies exhibiting favorable effects of MSC-ECM on young human BMSC stemness, proliferation, and osteogenic differentiation in 3D culture (Sadr et al., 2012; Antebi et al., 2015; Silva et al., 2020). Our findings in 3D iECM/silk scaffolds corroborate the stimulatory effects of iECM on osteogenesis, found with BMSCs from the three donors in 2D culture. Furthermore, our data suggest that iECM can enhance aged BMSC proliferation, whereas proliferation of young adult BMSCs in 3D scaffolds is overall higher and potentially limited by the available pore space.

To investigate whether the age and cellular reprogramming origin of the ECM-producing cells affected the osteogenic response, we compared the iECM layer with ECM layers engineered from BMSCs derived from donors under 30 years of age, and from BMSCs derived from donors over 70 years of age. Cells from two young adult and three aged donors were pooled to decrease the effect of inter-strain variation (Prewitz et al., 2013) and to obtain sufficient ECM from aged cells which were less proliferative. Contrary to prior studies demonstrating higher effects of young cell-ECM on proliferation and osteogenesis (Carvalho et al., 2021; Sun et al., 2011), we found only minor differences in the stimulatory effects of the three ECMs on aged BMSCs. This might be partially attributed to our selection of aged BMSCs, which exhibited relatively low senescence levels at the passages used (data not shown). Furthermore, the comparable outcomes between the three ECMs could potentially be attributed to our decellularization method, which was optimized for cell removal from ECM layers and 3D iECM/silk scaffolds according to the decellularization criteria (Crapo et al., 2011). It is known that the ECM affects cell differentiation responses by sequestering growth factors and modulating their proteolytic activation, as well as by interacting with cell surface receptors. (Suzawa et al., 1999; Dallas et al., 2002; Santra et al., 2002; Nili et al., 2003). In a prior study that demonstrated the effects of age of ECM-producing cells (Sun et al., 2011), the same chemicals but a shorter decellularization time was used, which might have better preserved the differences in ECM-anchored bioactive components. However, this prior study did not report the fulfillment of minimal criteria necessary to satisfy the intent of ECM decellularization (Sun et al., 2011).

Finally, we wanted to determine the extent of BMSC osteogenic responses when grown on iECM compared with single protein substrates. Prior studies showed that collagen type I substrate enhanced osteogenic differentiation of MSCs, mediated by the interaction with collagen-alpha 2 beta 1 integrin (Mizuno et al., 2000; Salasznyk et al., 2004; Kihara et al., 2006; Linsley et al., 2013). Fibronectin also exhibited a beneficial effect on MSC clonogenicity and proliferation via integrin alpha 6 (Nieto-Nicolau et al., 2020; Becerra-Bayona et al., 2012) and enhanced osteogenic differentiation (Linsley et al., 2013). In some prior studies, fibronectin exhibited a stronger stimulatory effect on MSC osteogenic differentiation compared with laminin, which was due to enhanced Akt and ERK signaling (Nieto-Nicolau et al., 2020; Linsley et al., 2013; Becerra-Bayona et al., 2012; Shen et al., 2019). ECMs from adipose tissue-derived MSCs and BMSCs, which were shown to specifically direct MSC differentiation, were found to contain similar proportions of collagens type I and VI, but differed in the extent of collagens type IV, V, and XII (Marinkovic et al., 2020). In our current study, a direct comparison between the iECM, fibronectin, and collagen substrates revealed a stronger effect of the iECM on aged BMSC proliferation and osteogenesis compared with single protein substrates. In order to elucidate the specific components of iECM responsible for its stimulatory effect on BMSC osteogenesis, further analyses of the iECM composition in conjunction with cell attachment-integrin blocking studies are required.

Interestingly, we found a reduction of adipogenesis of aged BMSCs on the iECM layer compared with single protein substrates. During the aging process, BMSCs ability to generate osteoblasts decreases, and the differentiation balance is shifted toward adipocyte formation (Stenderup, 2003; Wang et al., 2018). This adipogenic process may be strongly affected by the ECM composition (Marinkovic et al., 2020), as the decreased amount of collagen, for example, was shown to increase adipogenesis (Rodríguez et al., 2000). In a prior study, ECM layers have been shown to present elastic surfaces that were softer by entire orders of magnitude compared to tissue culture plastic or protein-coated glass substrate (Prewitz et al., 2013). Generally, substrates with rigidity close to collagenous bone were found to best support osteogenic differentiation of human MSCs (Chen and Jacobs, 2013). Therefore, further experiments would be necessary to determine whether the “rejuvenating” effect of our iECM on the BMSC osteo/adipogenic differentiation balance is mainly due to its composition, or additionally influenced by the mechanical properties of iECM.

A limitation of our study is that we have focused only on BMSCs, based on their common use in research and clinical studies of bone tissue engineering and regeneration. It would be interesting to evaluate whether a similar enhancement of osteogenic differentiation can be achieved for MSCs of other origins grown on iECM. Additionally, this study involved only one young adult BMSC donor and two aged BMSC donors for the testing of iECM effects on osteogenic capacity. As there is large inter-donor variability, precisely elucidating the effects (and potential differences) of iECM on BMSCs of young and aged donors would require testing BMSCs from additional donors. Finally, a detailed quantitative characterization of the iECM and specific components responsible for its stimulatory effects would provide further insight. It remains to be determined whether specific combinations of components as well as structure and mechanical properties of the iECM are contributing to the stimulatory effect on osteogenic differentiation of BMSCs.

## 5 Conclusion

In conclusion, we have shown that osteogenic differentiation of young adult human BMSCs can be strongly enhanced, and the compromised activity of aged human BMSCs can be partially restored by culturing them on an iECM layer engineered from hiPSC-MPs. We have also shown that our iECM enhanced the osteogenesis of BMSCs in 3D iECM/silk scaffolds, thus suggesting its potential use for bone regenerative therapies. Importantly, direct comparison of iECM to primary BMSC-derived ECM indicated a similar enhancement of BMSC osteogenic capacity. With hiPSCs representing a unique youthful, scalable human cell source for eventual clinical translation, tissue engineering strategies employing hiPSC-engineered ECM materials could potentially be developed to enhance bone regeneration in patients with delayed or failed bone healing, e.g., observed in older population.

## Data Availability

The raw data supporting the conclusion of this article will be made available by the authors, without undue reservation.
