# Supplementary material for: Engineering of extracellular matrix from human iPSC-mesenchymal progenitors to enhance osteogenic capacity of human bone marrow stromal cells independent of their age
Source: Front Bioeng Biotechnol. 2023 Aug 2;11:1214019. doi: 10.3389/fbioe.2023.1214019 (PMC10434254; doi:10.3389/fbioe.2023.1214019)
Supplement: Supplementary file 1 [file DataSheet1.docx]

# **Supplementary Figures­­**


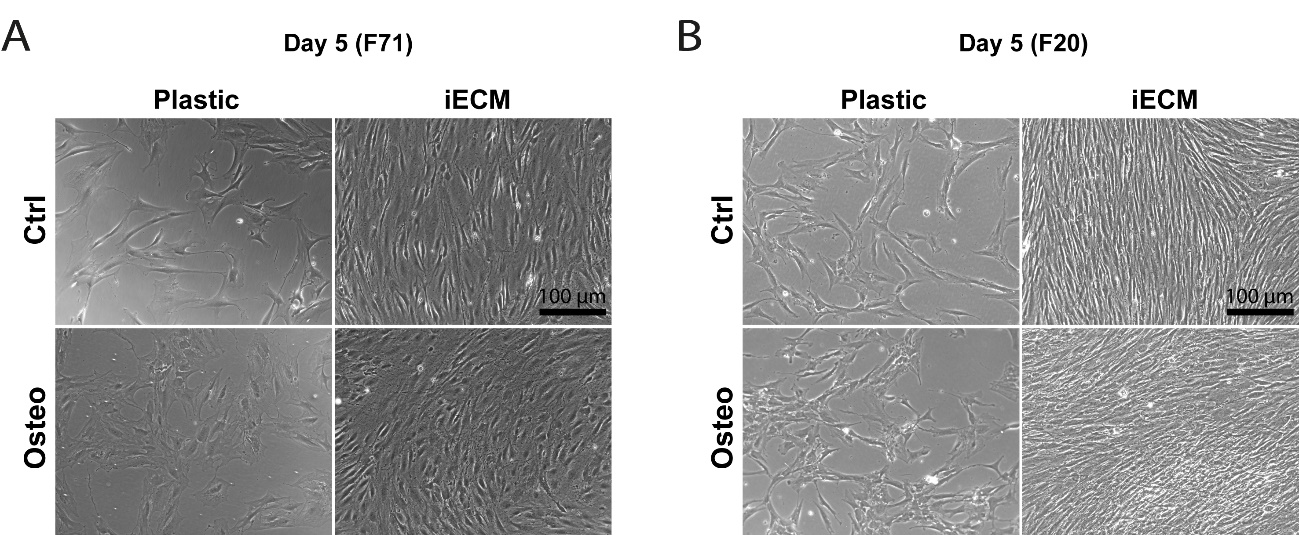


**Supplementary Figure 1: Enhanced proliferation of human BMSCs strains F71 (A) and F20 (B) on iECM layer.** Representative images of enhanced cell growth after 5 days of culture on the iECM layer as compared to standard tissue culture plastic in control and osteogenic media. Scale bars: 100 µm. Group labels: Ctrl - control medium, Osteo - osteogenic medium.


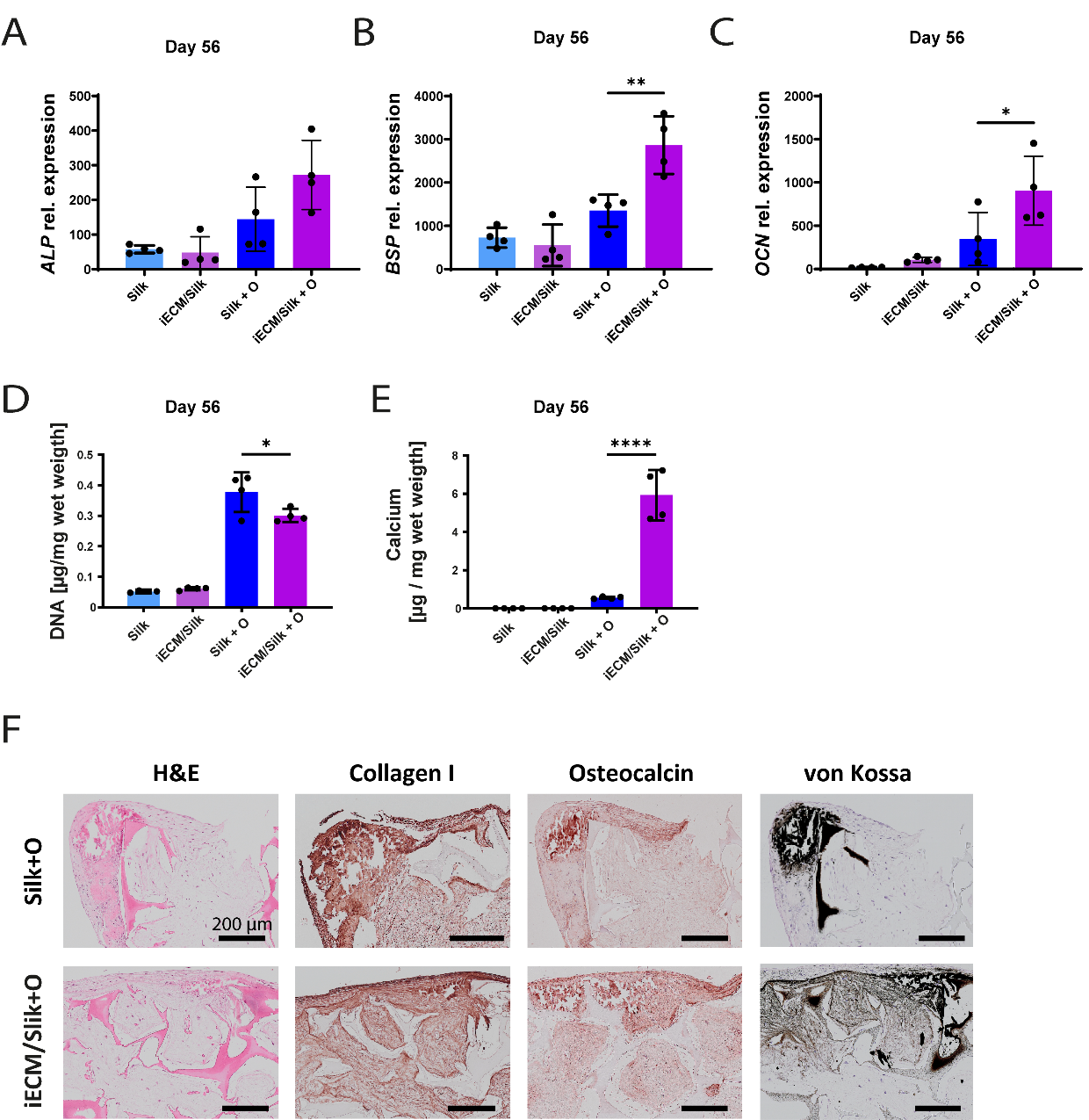


**Supplementary Figure 2: Enhanced osteogenic differentiation of young adult human BMSCs grown in 3D iECM/silk scaffolds**. **A-C)** Relative gene expression levels of osteogenic markers alkaline phosphatase (*ALP*, **A**), bone sialoprotein (*BSP*, **B**) and osteocalcin (*OCN*, **C**) after 56 days of culture. **D**) DNA content quantification after 56 days of culture. **E)** Calcium content quantification after 56 days of culture. **A-E**) Group labels: Silk - plain silk scaffold with control medium, iECM/silk – iECM/silk scaffold with control medium, Silk+O - plain silk scaffold with osteogenic medium, iECM/silk+O – iECM/silk scaffold with osteogenic medium. Data represents mean ± SD (n=4). Statistically significant differences between the groups were evaluated using a Kruskal–Wallis test, followed by Dunn’s multiple comparison test (A-E), and are marked with: *p < 0.05; **p < 0.01; ****p < 0.0001. **F)** Histological/immunohistochemical analyses of BMSCs cultured on plain silk scaffolds and iECM/silk scaffolds after 56 days of culture. Group labels: Silk+O - plain silk scaffold with osteogenic medium, iECM/silk +O - iECM/silk scaffold with osteogenic medium. Scale bars: 200 µm.


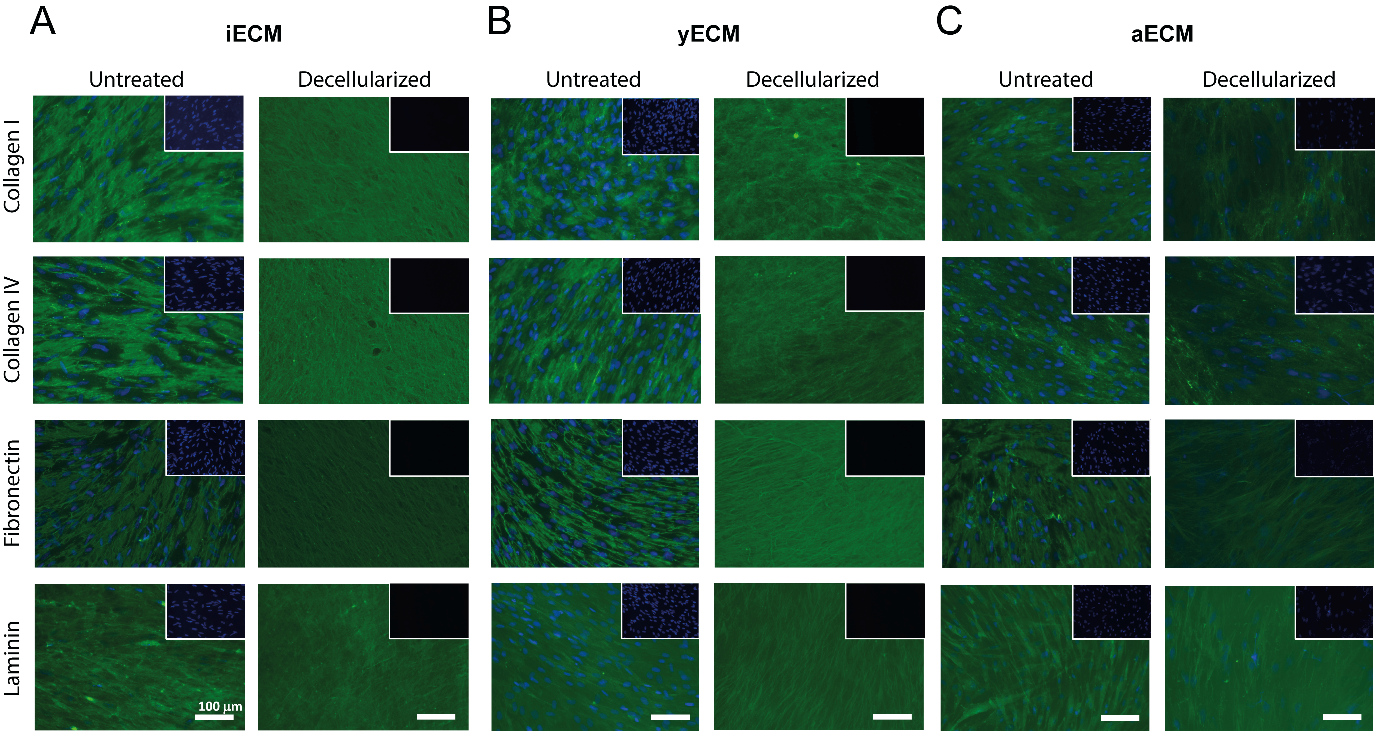


**Supplementary Figure 3: Characterization of ECM layers from cells of different ages before and after decellularization.** hiPSC-MP-ECM (iECM, **A**), young adult BMSCs-ECM (yECM, **B**) and aged BMSCs-ECM (aECM, **C**) were generated. Decellularized and untreated ECM layers stained positive for collagen type I, collagen type IV, fibronectin and laminin (green). Cell nuclei (blue) were present only in untreated iECM and yECM layers, whereas some remaining nuclear material was noted in aECM layer upon decellularization. Insets (top right of each picture) show negative staining controls. Scale bars: 100 µm.
